# Hydrolysis data for bis(4-cyanophenyl) phenyl phosphate including rate constants and activation parameters

**DOI:** 10.1016/j.dib.2019.104858

**Published:** 2019-11-22

**Authors:** V.E. Terekhov, V.V. Aleshkevich, E.S. Afanaseva, S.S. Nechausov, A.V. Babkin, B.A. Bulgakov, A.V. Kepman, V.V. Avdeev

**Affiliations:** a Lomonosov Moscow State University, Department of Chemistry, 119991, Leninskie Gory, 1-3, Moscow, Russia; b Institute of New Carbon Materials and Technologies, Russia

**Keywords:** Hydrolysis, Aryl phosphate, Rate constant, Activation parameters, Phthalonitrile resins, Reactive diluent

## Abstract

Hydrolysis data for Bis(4-cyanophenyl) phenyl phosphate (**CPP**), introduced as a reactive diluent for phthalonitrile monomers, under pH 4, 7 and 10 are presented. Conversion/time plots collected by HPLC analysis, typical chromatograms and NMR spectra of the substrate and the reaction products are given. Pseudo-first order rate constants are determined for **CPP** at 25, 50 and 80 °C. Activation parameters were calculated from Arrhenius equation.

**Table:** Specifications Table

Subject area	*Chemistry*
More specific subject area	*Phthalonitrile resins*
Type of data	*Tables, graph, figure*
How data was acquired	Agilent 1260 chromatographer (column ZORBAX Eclipse Plus C18); *Bruker Avance 600 at* 162 MHz *for*^*31*^*P NMR with DMSO-d6 as solvent*
Data format	*Analyzed*
Experimental factors	*Samples were diluted in acetonitrile/buffer solution -50/50 (vol) in a concentration of* 1 mg/ml*, heated on a thermostat for determined time periods and analyzed with HPLC.*
Experimental features	*Withdrawn aliquots were frozen with liquid nitrogen and unfrozen prior to analysis.*
Data source location	*Moscow, Russian Federation*
Data accessibility	*Analysed data are available with the article. All raw data have been deposited in the public repository.* *Repository name: Mendeley Data* *Data identification number:*http://doi.org/10.17632/3fmv6p3smk.1 *Direct URL to data:*https://data.mendeley.com/datasets/3fmv6p3smk/1
Associated article	*V. E. Terekhov, V. V. Aleshkevich, E. S. Afanaseva, S. S. Nechausov, A. V. Babkin, B. A. Bulgakov, A. V. Kepman, and V. V. Avdeev. Bis(4-cyanophenyl) phenyl phosphate as viscosity reducing comonomer for phthalonitrile resins. Reactive and Functional Polymers,* 2019*, V. 139, P. 34–41.* DOI: 10.1016/j.reactfunctpolym.2019.03.010

**Table:** 

**Value of the Data** • The presented data gives new insights on hydrolysis of aryl-phosphoric esters and defines the limits of **CPP** applications. • Chemists and engineers working with thermosetting resins, as well as physical chemists studying hydrolysis can benefit from these data. • The presented data can be used to generalize hydrolysis behavior of aryl-phosphates and define the suitability of **CPP** for engineering purposes. • The data can help developing easy-processable highly heat-resistant thermosetting resins. • Only one-step hydrolysis was detected under all considered pH values indicating only 4-cyanophenol elimination.

## 1 Data

**Fig. 1:**
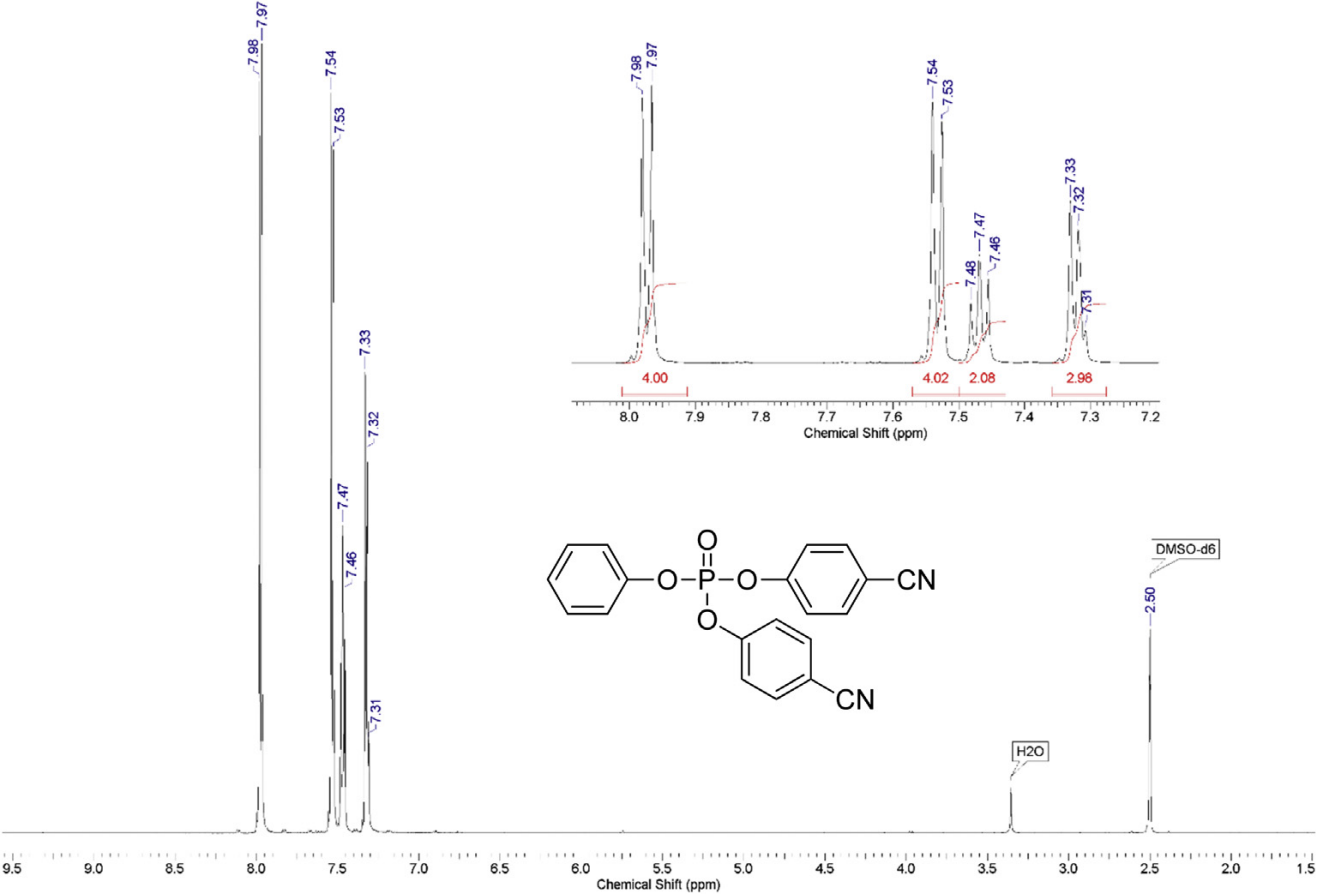
^1^H NMR spectrum of bis(4-cyanophenyl) phenyl phosphate.

**Fig. 2:**
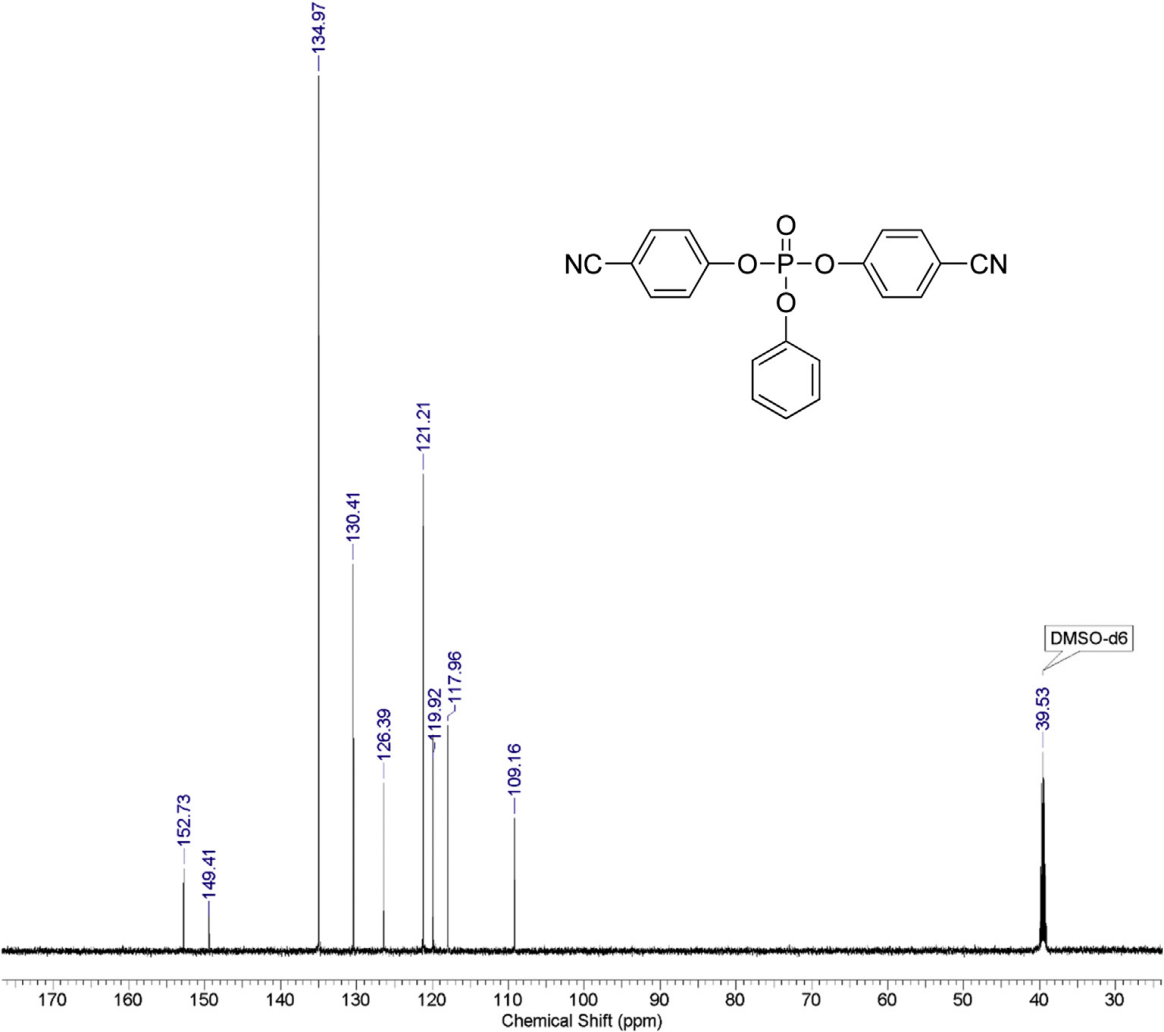
^13^C NMR spectrum of bis(4-cyanophenyl) phenyl phosphate.

**Fig. 3:**
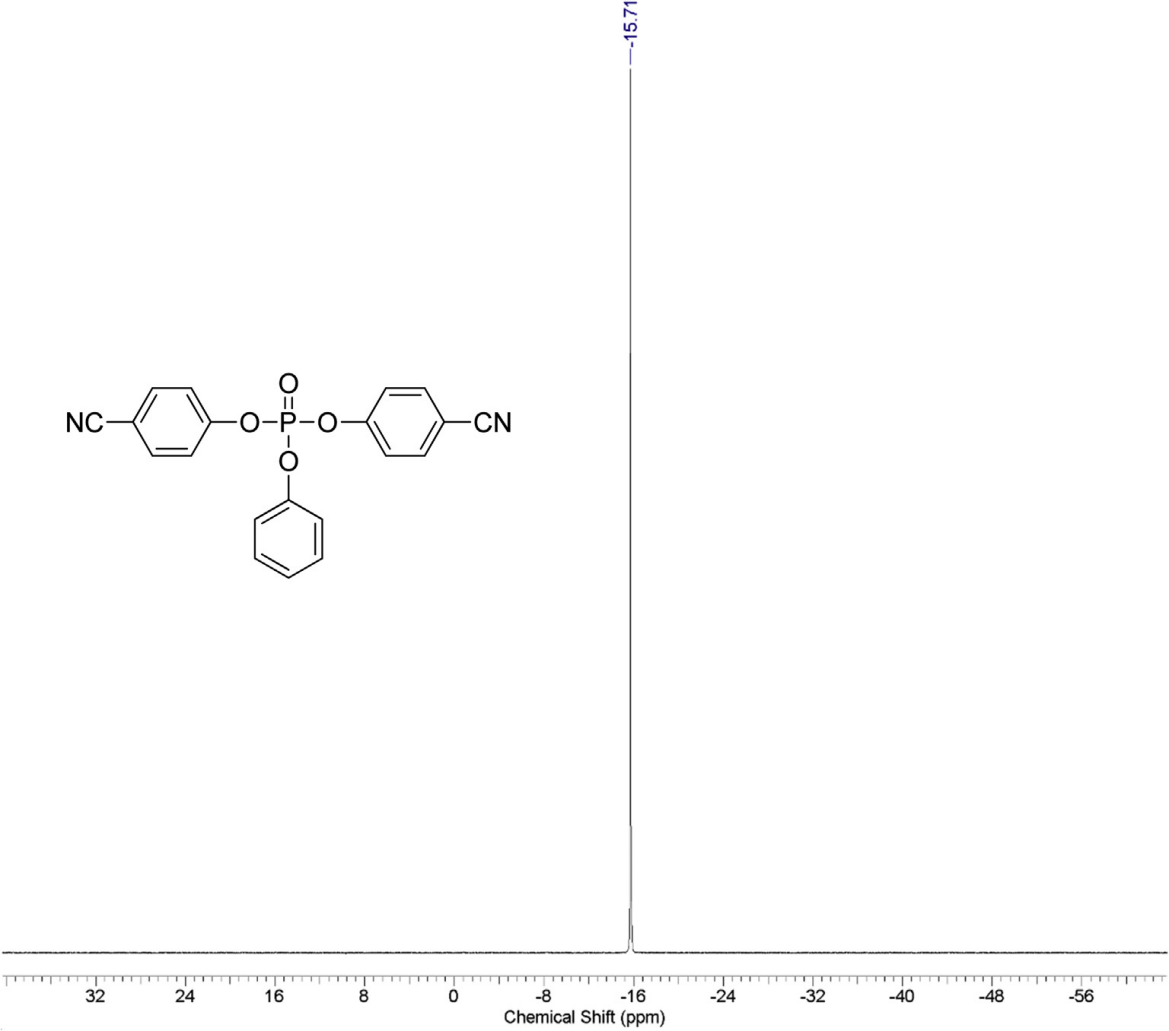
^31^P NMR spectrum of bis(4-cyanophenyl**)** phenyl phosphate.

The dataset contains raw NMR-spectroscopy data for **CPP** (can be opened with e.g. ACDLabs Spectrus processor), analyzed ^1^H, ^13^C and ^31^P spectra (Fig. 1, Fig. 2, Fig. 3), raw chromatograms of the HPLC hydrolysis study including initial chromatograms of **CPP**, 4-cyanophenol and phenol, and chromatograms obtained during the hydrolysis study, as well as calculated rate constants and activation parameters (Table 2, Table 3), concentration/time plots (Fig. 5, Fig. 6, Fig. 7), analyzed ^1^H, ^13^C, and ^31^P spectra of the hydrolysis products (Fig. 8, Fig. 9, Fig. 10).

**Table 1:** Elution program applied for LC analysis.

Time, min	Acetonitrile, %	H_2_O, %
0–5	55	45
5–25	55–98	45–2
25–28	98	2
28–33	98–55	2–45
33–35	55	45

**Table 2:** Rate constants and conversion for hydrolysis of CPP at various pH values.

T, °C	pH 4		pH 7		pH 10	
	k, s^−1^	Conversion, %	k, s^−1^	Conversion, %	k, s^−1^	Conversion, %
25	1.20 × 10^−6^	20 (48 h)	9.72 × 10^−6^	80 (48 h)	1.36 × 10^−4^	91 (5 h)
35	2.70 × 10^−6^	40 (48 h)	2.47 × 10^−5^	100 (48 h)	4.28 × 10^−4^	100 (3 h)
50	7.31 × 10^−6^	68 (48 h)	8.33 × 10^−5^	100 (12 h)	2.15 × 10^−3^	97.5 (30 min)
60	1.89 × 10^−5^	73 (24 h)	2.21 × 10^−4^	100 (6 h)	4.94 × 10^−3^	99 (15 min)
80	6.45 × 10^−5^	58 (4 h)	9.69 × 10^−4^	100 (1.5 h)	–	100 (3 min)

**Table 3:** Activation parameters of **CPP** hydrolysis.

pH	Е_А_, kcal/mole	А, s^−1^
4	15.22	1.73 × 10^5^
7	17.52	6.87 × 10^7^
10	20.43	1.31 × 10^11^

**Fig. 4:**
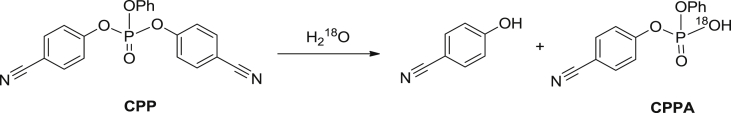
Established hydrolysis reaction of CPP under pH = 4, 7 and 10.

**Fig. 5:**
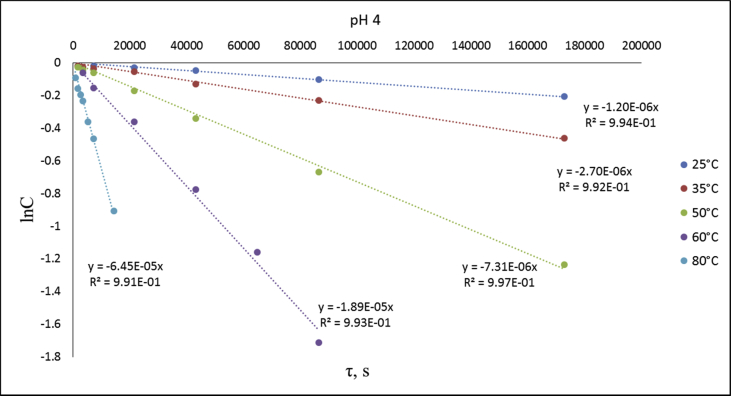
lnC-time plots for phthalonitrile CPP hydrolysis at different temperatures and pH 4.

**Fig. 6:**
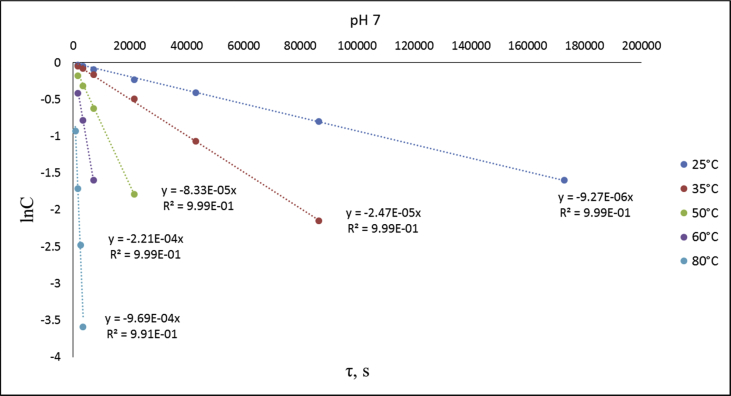
lnC-time plots for phthalonitrile CPP hydrolysis at different temperatures and pH 7.

**Fig. 7:**
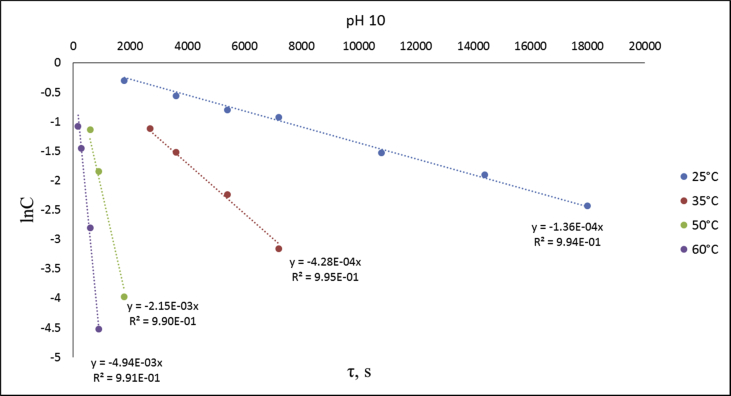
lnC-time plots for phthalonitrile CPP hydrolysis at different temperatures and pH 10.

**Fig. 8:**
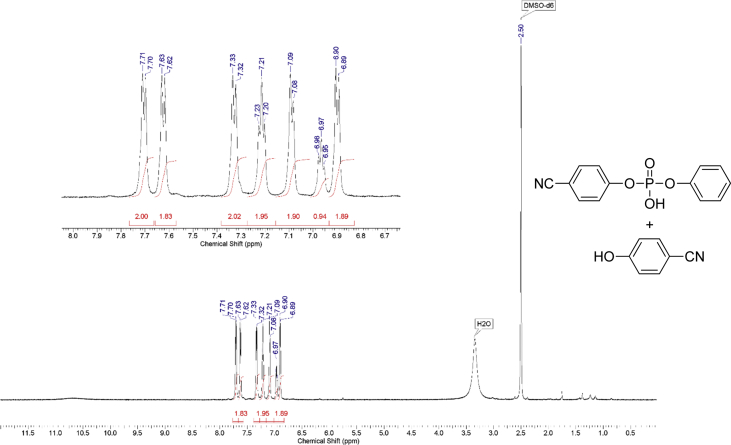
^1^H NMR spectrum of products of hydrolysis.

**Fig. 9:**
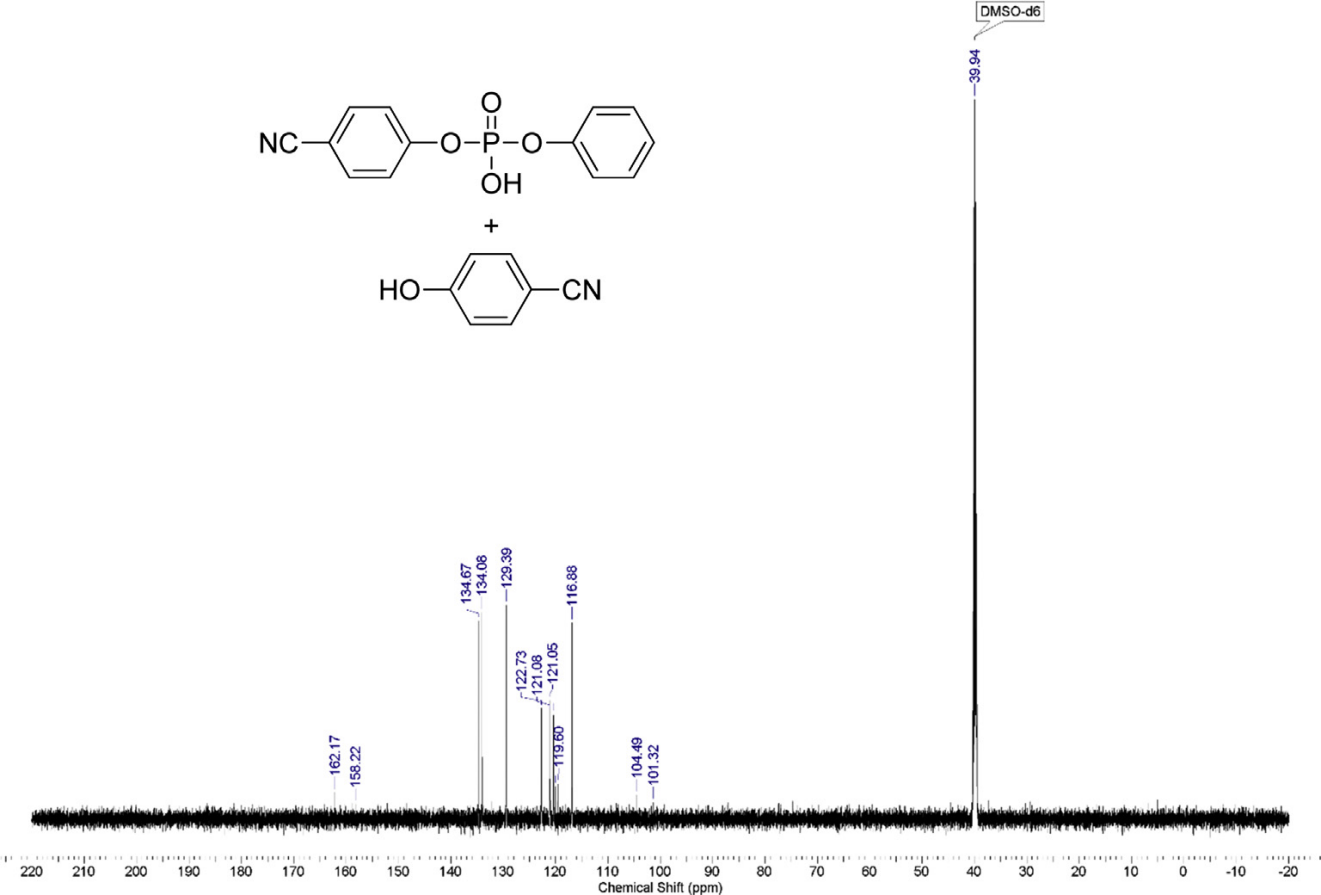
^13^C NMR spectrum of products of hydrolysis.

**Fig. 10:**
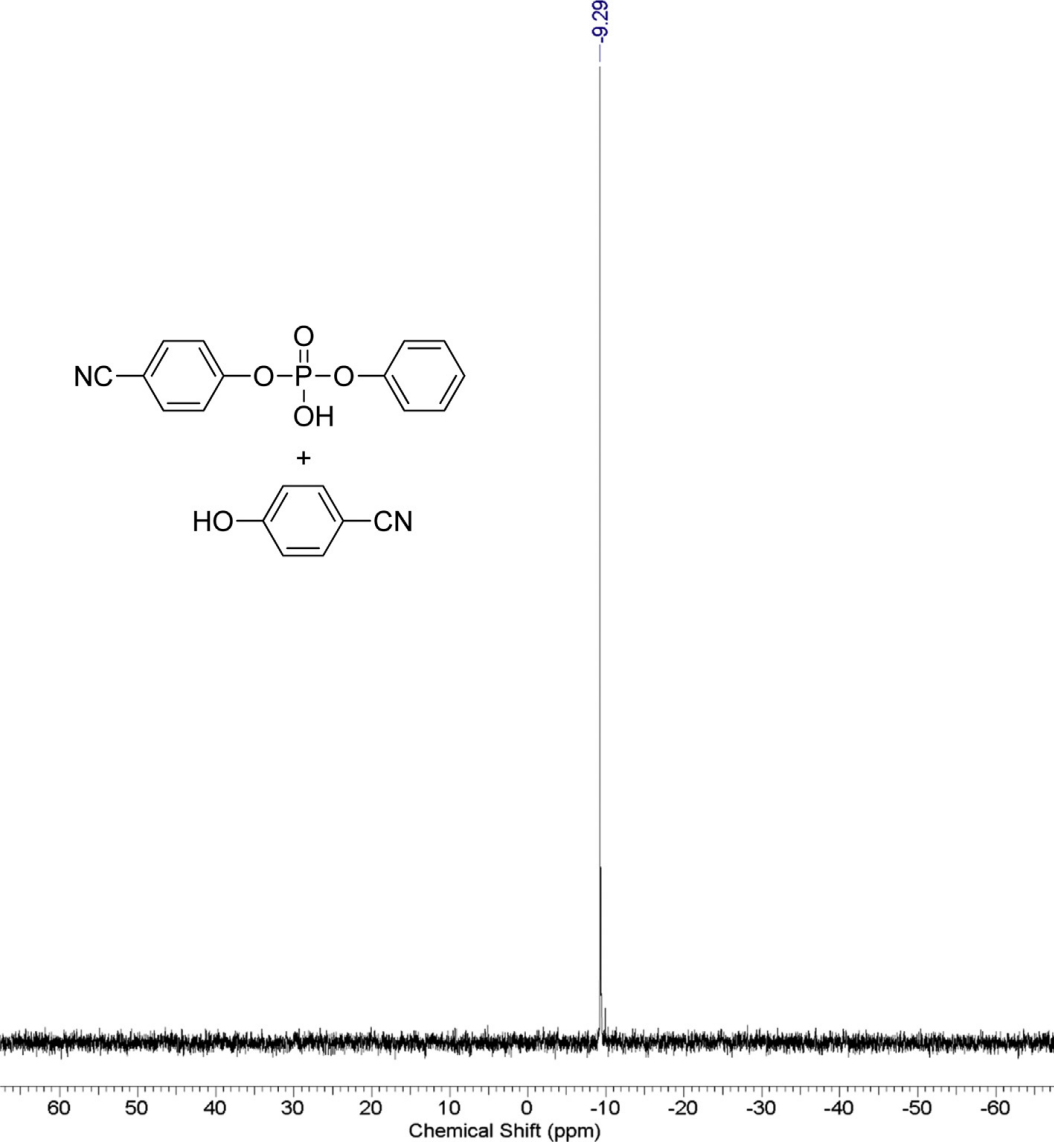
^31^P NMR spectrum of products of hydrolysis.

## 2 Experimental design, materials and methods

### 2.1 Materials

4-cyanophenol and phenyldichlorophosphate were obtained from Sigma Aldrich and used as received. Toluene and pyridine were obtained from Chimmed (Moscow, Russia). Toluene was distilled over sodium metal and pyridine was distilled over CaH_2_, according to standard procedures [1].

Acetonitrile (HPLC grade) was obtained from Sigma Aldrich and used as received. Buffer solutions with pH 4, 7 and 10 were purchased from Panreac Applichem. According to the product description, the pH of these buffers was maintained in a temperature ranging from 20 °C to 100 °C.

### 2.2 Monomer **CPP** synthesis

1 g (0.008 mol) of anhydrous potassium bromide and 6.32 g (0.08 mol) of pyridine were added to a solution of 9.52 g (0.08 mol) of 4-cyanophenol in 50 mL of dry toluene. The mixture was stirred at 70 °C for 30 minutes under an inert atmosphere of argon in a 100 ml three-neck flask. Then, 8.44 g (0.04 mol) of phenyl dichlorophosphate was added dropwise. The mixture was stirred at 70 °C for 24 hours under an inert atmosphere. After that, the reaction mixture was cooled, filtered from pyridine hydrochloride and the filtrate was washed with water (3×30 mL). The water layer was washed with toluene (3×10 mL) and the combined organic phases were dried over anhydrous sodium sulfate. The solution was then evaporated using a rotary evaporator and the residue was dried at 80 °C for 2 hours under 5 mmHg. 14.25 g of yellowish solid was obtained at a yield of 95%. According to the NMR ^1^H spectrum, the product purity was defined as 95%. To obtain a purity greater than 99%, flash chromatography on silica gel was carried out (eluent – CH_2_Cl_2_:MeOH 29:1). 13.47 g (89.5% of total yield) of the product was obtained as a white solid after evaporation of the solvent. ^1^H, ^13^C and ^31^P NMR spectra are presented in Fig. 1, Fig. 2, Fig. 3.

^1^H NMR (600 MHz, DMSO-d6) δ ppm 7.21–7.39 (m, 3H), 7.47 (t, J = 7.79 Hz, 2H), 7.53 (d, J = 8.44 Hz, 4H), 7.98 (d, J = 8.53 Hz, 4H)
^13^C NMR (151 MHz, DMSO-d6) δ ppm 109.14, 117.96, 119.93 (d, J = 4.42 Hz), 121.23 (d, J = 5.53 Hz), 126.39, 130.41, 134.97, 149.42 (d, J = 7.74 Hz), 152.74 (d, J = 6.64 Hz)
^31^P NMR (243 MHz, DMSO-d6) δ ppm −15.71.

Anal. Calcd. for C_20_H_13_N_2_O_4_P: C 63.84, H 3.48, N 7.44, Found C 63.81, H 3.53, N 7.40.

### 2.3 Methods

To study the hydrolysis reaction HPLC analysis was carried out for the collected samples with an Agilent 1260 chromatographer equipped with a column ZORBAX Eclipse Plus C18 (Т_column_ = 30°С; flow rate – 0.8 mL/min). The gradient elution method was used, and the elution program is presented in Table 1. Agilent ChemStation software was used to develop chromatograms.

### 2.4 Sample preparation and hydrolysis study

To obtain the initial solution monomer **CPP** was diluted in acetonitrile to reach concentration 2 mg/ml. Then, the initial solution (10 ml) and buffer solution (10 ml) were heated to the aimed temperature (25, 50 or 80°С) and mixed under severe stirring with a magnetic bar. From this moment the reaction time was measured. The reaction mixture was held in a water bath under stirring and aliquots (1 ml) were withdrawn in certain time intervals, then sealed in 1.5 ml glass vials equipped with septum heads and frozen in liquid nitrogen to prevent any chemical processes. Samples were unfrozen immediately before HPLC analysis.

In the case of the hydrolysis study under pH 10, withdrawn samples were poured into 10 μl of concentrated HCl and shaken to prevent further hydrolysis. Then, they were immediately frozen in liquid nitrogen as reported for the hydrolysis of phthalonitrile phosphate and phosphonate [2].

Two series of aliquots were selected to obtain the average values of the peak areas in the chromatograms.

### 2.5 HPLC-analysis

Series of solutions of **CPP**, 4-cyanophenol and phenol with concentrations 1.50, 1.25, 1.00, 0.75, 0.50, 0.25 mg/ml were prepared for each substance by subsequent dilution of the initial solutions (2 mg/ml) with acetonitrile for LC calibration. Concentrations of the studied compounds were determined based on calibration by automatic analysis with Agilent ChemStation software.

An assumption was made for the results' interpretation: hydrolysis of the monomer **CPP** was considered as a pseudo-first-order reaction due to the sufficiently higher water concentration in experimental than the substrate's concentrations. It was established that hydrolysis passed only by 4-cyanophenol elimination [3] (Fig. 4). Concentration (lnC) versus time plots were obtained (Fig. 5, Fig. 6, Fig. 7). Pseudo-first order constants and activation parameters were calculated (Table 2, Table 3). ^1^H, ^13^C and ^31^P NMR spectra of hydrolysis' products are presented on Fig. 8, Fig. 9, Fig. 10.
